# Radiological signs of stone impaction add no value in predicting spontaneous stone passage

**DOI:** 10.1007/s00240-024-01604-0

**Published:** 2024-08-06

**Authors:** Marcin Popiolek, Mats Lidén, Petros Georgouleas, Klara Sahlén, Pernilla Sundqvist, Johan Jendeberg

**Affiliations:** 1 Department of Urology, Faculty of Medicine and Health, Örebro University, 701 85 Örebro, Sweden; 2 Department of Radiology, Faculty of Medicine and Health, Örebro University, Örebro, Sweden; 3 Department of Surgical Sciences, Radiology, Uppsala University, Uppsala, Sweden

**Keywords:** Ureteral stone, Passage, Impaction, Prediction

## Abstract

Stone size and location are key factors in predicting spontaneous stone passage (SSP), but little attention has been paid to the influence of radiological signs of stone impaction (RSSI). This research aims to determine whether RSSI, alongside stone size, can predict SSP and to evaluate the consistency of ureteral wall thickness (UWT) measurements among observers. In this retrospective study, 160 patients with a single upper or middle ureteral stone on acute non-enhanced computed tomography (NCCT) were analysed. Patient data were collected from medical records. Measurements of RSSI, including UWT, ureteral diameters, and average attenuation above and below the stone, were taken on NCCT by four independent readers blind to the outcomes. The cohort consisted of 70% males with an average age of 51 ± 15. SSP occurred in 61% of patients over 20 weeks. The median stone length was 5.7 mm (IQR: 4.5–7.3) and was significantly shorter in patients who passed their stones at short- (4.6 vs. 7.1, *p* < 0.001) and long-term (4.8 vs. 7.1, *p* < 0.001) follow-up. For stone length, the area under the receiver operating characteristic curve (AUC) for predicting SSP was 0.90 (CI 0.84–0.96) and only increased to 0.91 (CI 0.85–0.95) when adding ureteral diameters and UWT. Ureteral attenuation did not predict SSP (AUC < 0.5). Interobserver variability for UWT was moderate, with ± 2.0 mm multi-reader limits of agreement (LOA). The results suggest that RSSI do not enhance the predictive value of stone size for SSP. UWT measurements exhibit moderate reliability with significant interobserver variability.

## Introduction

Ureter calculi are a common cause of emergency visits and a substantial burden on both the affected and healthcare systems. Although most ureteral stones pass spontaneously with low morbidity (75–90%), some patients may require multiple interventions, which can be accompanied by various treatment-related complications [1]. Furthermore, ureteral stone-related morbidity includes a risk of septicaemia, kidney failure and hypertension. These complications can significantly impact the patient’s quality of life, imposing substantial limitations [2].

According to the European Association of Urology (EAU) Urolithiasis Guidelines, stones with a low likelihood of spontaneous passage should be considered for early intervention. It is well established that stone size is an excellent predictor of spontaneous stone passage (SSP), with over 90% accuracy as a single predictor. However, the EAU guidelines panel concludes that no exact cut-off value for stone size can be provided owing to a lack of evidence [3]. Thus, identifying additional reliable predictors of SSP is crucial for determining appropriate management strategies, optimising patient care and avoiding unnecessary interventions.

While previous studies have explored several predictors of stone passage, such as stone size, location and composition, limited attention has been given to the role of radiological signs of stone impaction (RSSI) in predicting SSP [4–6]. Including ureter dilatation, ureteral wall thickness and ureter attenuation above and below the stone, RSSI visualised on CT imaging may indicate stone impaction and the associated inflammatory response [7–9]. Ureteral wall thickness (UWT) and other ureter-related factors have emerged as potential predictors of SSP, reflecting the dynamic interaction between the stone and the ureter during its passage [10–12]. Investigation of RSSI may provide additional valuable insights into the mechanisms underlying stone passage and potentially facilitate risk stratification and tailored treatment approaches. Yet, it has not been studied whether RSSI can add any significant value to stone size in predicting SSP. Moreover, according to the most recent studies, UWT can be sufficiently measured on non-contrast-enhanced computed tomography (NCCT) [7–9]. However, no standardised method has yet been introduced or validated.

The present study aimed to assess whether RSSI, in addition to stone size, provide information for predicting SSP and to estimate interobserver variability in UWT measurements.

## Material and methods

### Study population

Ethical approval was obtained from the Swedish Ethical Review Authority (No.2014/136).

**Fig. 1:**
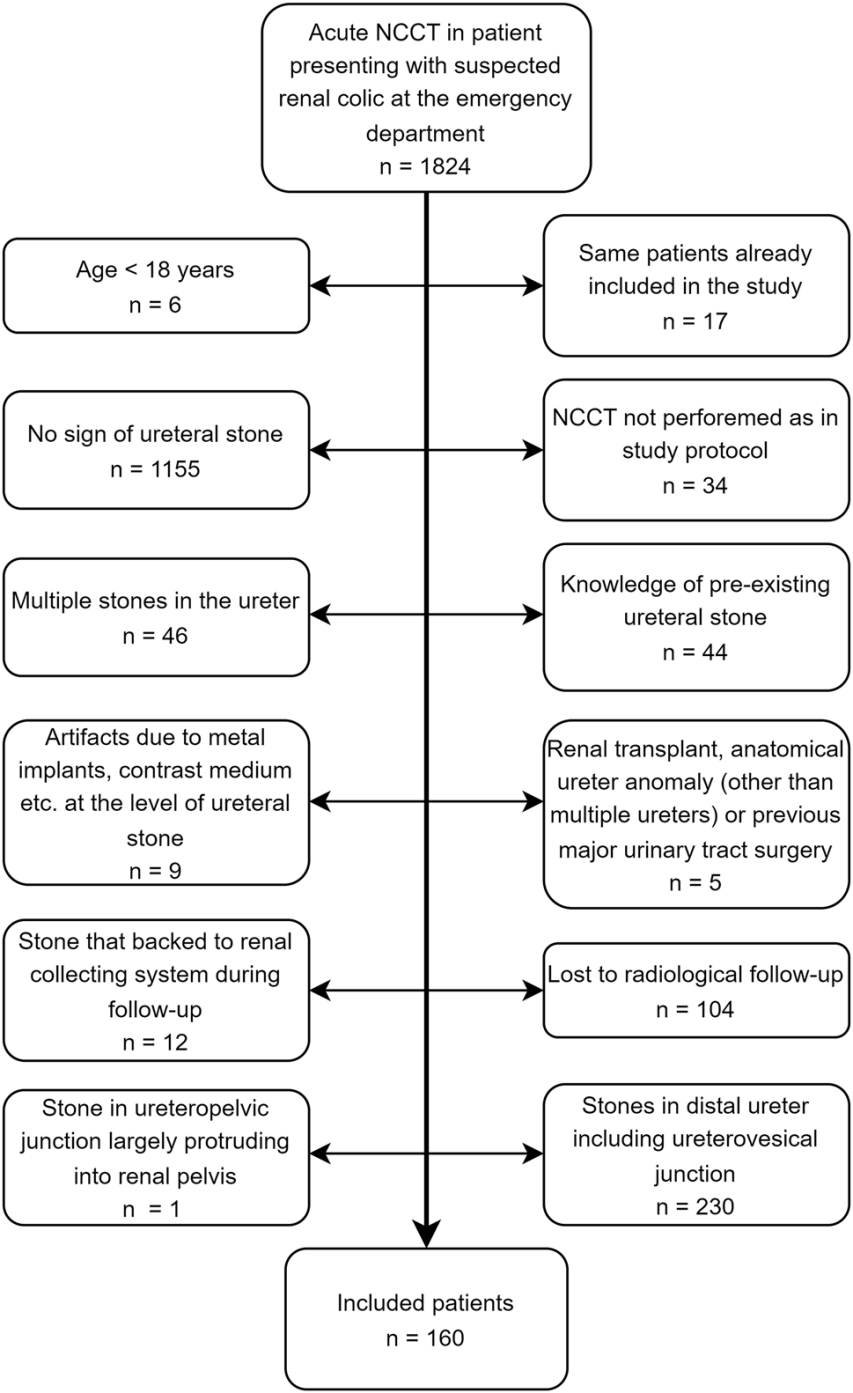
Flowchart showing exclusion criteria with numbers

The study was performed using a previously reported stone passage databank, in which stone expulsion rates for the whole cohort in relation to stone size and location, but not RSSI, were reported [6]. A retrospective review was carried out of 1,824 consecutive patients who presented at our emergency department with flank pain and underwent NCCT performed between April 2012 and September 2014. The inclusion criterion was a solitary ureteral stone > 2 mm in diameter in the axial plane. Exclusion criteria inclusive numbers are shown in the flowchart in Fig. 1.

### Sample-size analysis

We calculated the required sample size with regard to UWT based on the findings from previous studies, in terms of both the proportion of the non-events and the estimated standard deviation of UWT in the population. To achieve 80% power with an effect size of 1-mm difference in UWT between the event and non-event groups, a sample size of at least 150 subjects was needed. Because of the high probability of SSP in distal ureteral stones, only stones in the upper and middle ureter were included, resulting in a study population of 160 subjects.

### CT protocol

The CT examinations were intermediate-dose non-contrast enhanced scans performed on two different CT scanners: 67 patients were examined using a 40-detector row CT scanner (Brilliance, Philips Medical Systems, Best, The Netherlands) with a low-dose NCCT protocol for the urinary tract (120 kV, 70 mAs/slice, CTDI 4.9 mGy), and 93 patients were examined with a 2 × 128-channel scanner (Somatom Definition Flash, Siemens, Erlangen, Germany) (120 kVp, 70 mAs/slice CTDI 4.7). Manual measurements were performed on both axial 1-mm slices and 3-mm axial, sagittal and coronal reformats, which were generated in the main axes of the patient.

### Patient data

Patient-related data such as age, sex, stone laterality and C-reactive protein (CRP) at diagnosis or interventions were retrieved from the medical records. Stone-related data were obtained from CT scans using the integrated PACS measurement tool (Sectra IDS7, Linköping, Sweden).

### Radiological evaluations

#### Stone size

Stone size was measured according to the methodology previously described by Jendeberg et al*.* (i.e. independently by three readers in the axial, coronal and sagittal reformations in a soft-tissue window) [6]. Stone length was defined as the largest of the three reformation measurements. The mean value of three readers was used. Our cohort included only upper and middle ureteral stones, which were defined as being located in the ureter segment between the ureteropelvic junction and the lower edge of the sacroiliac joint.

#### Radiological signs of stone impaction

**Fig. 2:**
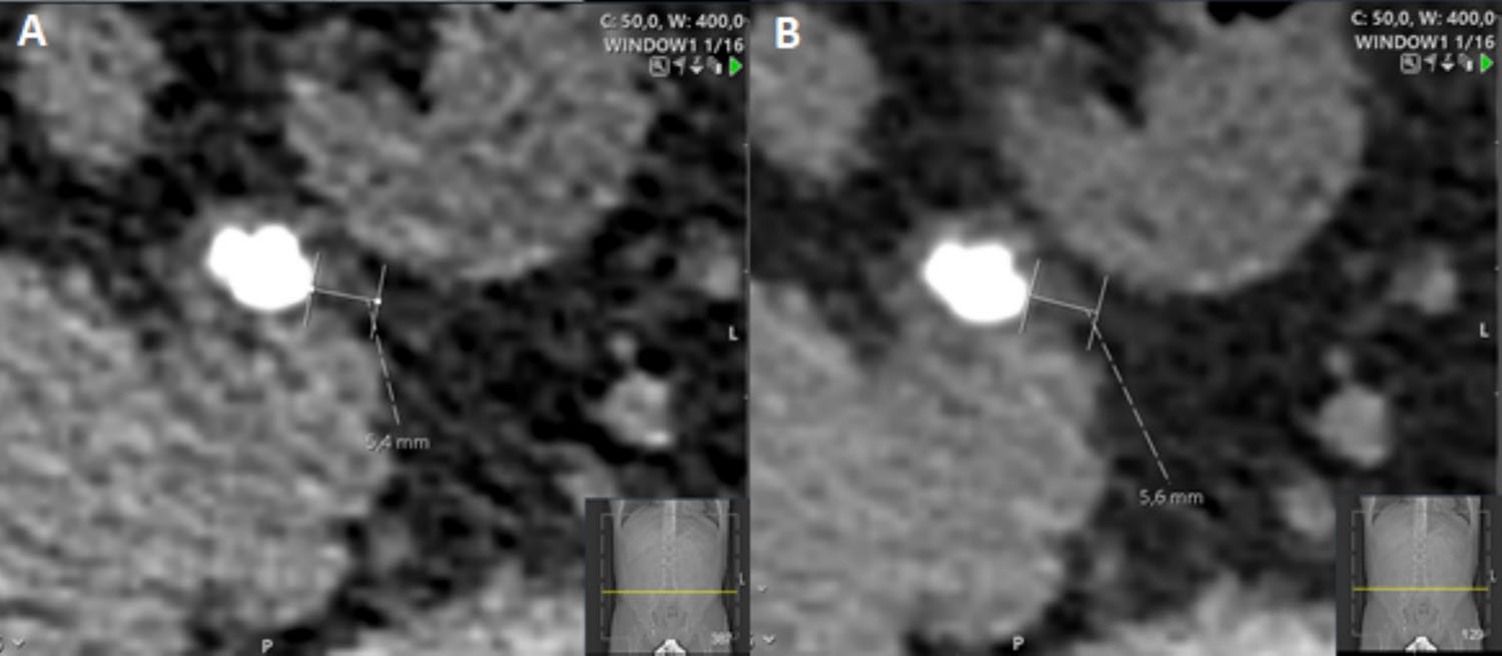
UWT measured on 1-mm axial slice (**A**) and 3-mm axial slice (**B**)

UWT was measured at the spot of greatest soft-tissue thickness (ureteral wall + periureteral oedema) both on axial 1-mm slices and 3-mm reformations around the stone circumference at the level of its largest axial diameter (Fig. 2).

**Fig. 3:**
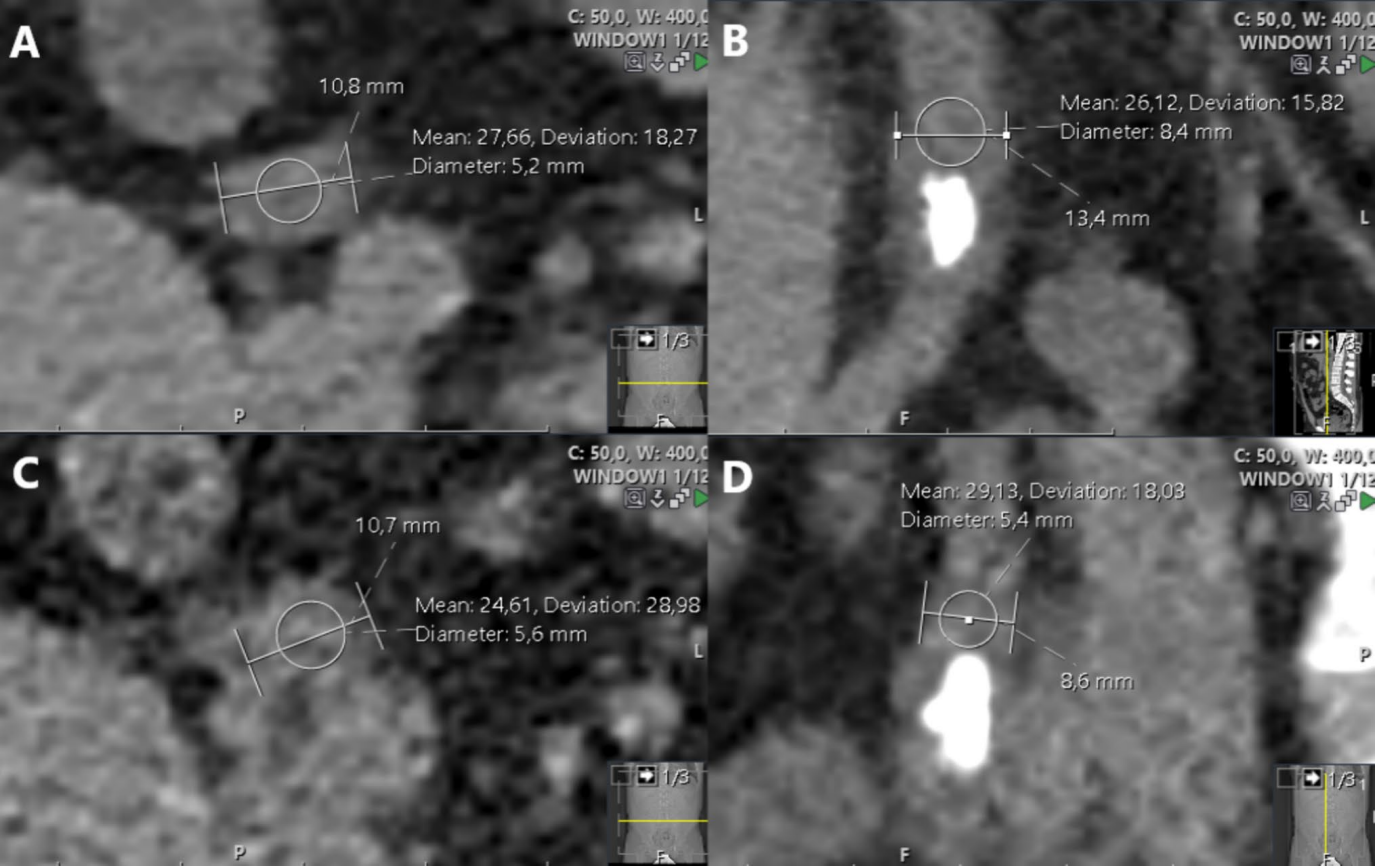
UAAS and USAS measured on all reformations: **A** axial 3 mm; **B** coronal 3 mm; **C** axial 1 mm; **D** sagittal 3 mm. UDAS and UDBS were measured analogously but below the stone

Ureter diameter (UD) was measured one slice below (UDBS) and above (UDAS) the stone on all reformations (including 1- and 3-mm axial slices) at its widest place. At the same spot as the UD, the average ureter attenuation was measured both above (UAAS) and below (UABS) the stone, manually placing a circular region of interest (ROI) within the ureter covering up to 2/3 of the surface in all reformations (Fig. 3).

UWT, UDs, UAAS and UABS were measured in a standardised soft-tissue window (L50/W400) by four independent readers, of which two were radiologists (JJ, KS) and two were urologists (MP, PG). The readers were not aware of the spontaneous passage status at the time of measurement. A median value of all readers was used for further analysis. Stone length, UWT and UDs were reported in millimetres to one decimal place.

The presence of hydronephrosis was independently graded as 0–3 (0 = no, 1 = mild, 2 = moderate, 3 = severe) by MP. Renal pelvis diameter (RPD) was measured on 1-mm axial slices between the anterior and posterior wall at its widest place (anteroposterior diameter) by one reader (MP). The presence of a rim sign (i.e. a soft tissue rim around the stone on the axial planes) was assessed by two readers (MP and JJ) independently, and only concordant assessments were taken for further analysis as a positive rim sign.

### Study endpoints

All radiological examinations were reviewed up to 26 weeks after diagnosis with regard to SSP or intervention. SSP was defined as absence of a stone on follow-up imaging after conservative treatment including analgesics and/or medical expulsive therapy (MET), without any need for surgical intervention, such as shock wave lithotripsy (SWL), ureteroscopy (URS) or drainage (double pigtail catheter or nephropyelostomy tube). Patients who underwent surgical interventions were included in the analysis as failed SSP. However, no standardised protocol for indication to surgical intervention was utilised due to retrospective nature of this study. The decision to intervene surgically was made individually by the responsible urologist based on best clinical practice and current guidelines. Follow-up imaging in the SSP group included intravenous urography (IVU) (n = 69), NCCT (n = 19) or contrast-enhanced CT (CECT) (n = 9). According to the local routine, follow-up imaging was first performed after 4–6 weeks if the patient qualified for conservative treatment. Additional follow-up imaging was usually advised after 4–6 weeks if the stone was present at the first control and there was still no indication of a need for surgical intervention.

As described previously, passage rates in the short and long term were determined [6]. A short-term subgroup was identified, including patients with conservative follow-up imaging or surgical intervention within 28 ± 14 days. Similarly, the long-term outcome group included all the patients who were managed conservatively or with surgical intervention during the period of up to 140 days (20 weeks).

### Statistics

The statistical analysis was performed using IBM SPSS v27.0.1.0 (SPSS Inc., Chicago, IL, USA). Between-group comparisons were performed using Pearson’s chi-square or Fisher’s exact test for quantitative variables and Student’s *t*-test or the Mann–Whitney *U* test for continuous variables. Correlations between predictors were assessed with the Pearson or Spearman correlation coefficients. Because of high correlation (|*r*| > 0.5) and no significant difference in prediction accuracy between UWT, UAAS, UABS, UDAS and UDBS measured on different reformats, only measurements performed on 1-mm axial slices were selected for further analysis and are reported in this article. To detect potential multicollinearity among continuous variables, we calculated the variance inflation factor (VIF) prior to multivariable analysis. VIF values > 5 were considered to indicate a high multicollinearity. Multivariable analysis was conducted with binary logistic regression using SSP as the dependent variable. Receiver operating characteristic (ROC) curves were calculated for stone length separately and in combination with stone impaction variables (UDAS, UDBS and UWT) using probabilities from logistic regression. Furthermore, to determine the reproducibility of the measurements, we investigated both reliability and inter-observer agreement. Reliability was assessed by computing the intra-class correlation coefficient (ICC) using analysis of variance (ANOVA; two-way mixed model with absolute agreement). Values close to 1 indicate high reliability. Agreement plots were created in which the difference between the reader’s measurement and the mean measurement (*y*-axis) was plotted against the mean measurement [13]. A two-sided *p* < 0.05 was considered statistically significant.

## Results

Demographics and baseline clinical data are summarised in Table 1. Of all the patients, 112 (70%) were males, and the mean age was 51 ± 15 years. Baseline stone characteristics, together with a comparison of the radiological parameters between the stone passage and non-passage groups, are presented in Table 2. In the 20-week follow-up, SSP was observed in 97 patients (61%), and 61 patients (38%) needed intervention. The median stone length was 5.7 mm (IQR: 4.5–7.3) and differed significantly between the passage and the non-passage groups in both short-term (4.6 vs. 7.1, *p* < 0.001) and long-term (4.8 vs. 7.1, *p* < 0.001) follow-up. The median UWT was 2.4 (IQR: 1.9–3.3) for the whole cohort, and there was a statistically significant difference between passage and non-passage in both the short-term (*p* = 0.003) and long-term groups (*p* = 0.001). There was a lower proportion of rim signs in the passage versus the non-passage groups regarding both short- and long-term outcomes, with 14% versus 25% (*p* = 0.2) and 9% versus 25% (*p* = 0.008), respectively. The median UDAS and UDBS (measured on axial 1-mm slices) were 7.5 mm (IQR: 6.3–9.8) and 6.3 mm (IQR: 5.3–7.7), respectively. There was a significant difference between UDAS and UDBS in patients with passage and non-passage in both short and long-term follow-up (UDAS: *p* < 0.001; UDBS: *p* < 0.001).

**Table 1:** Demographics and clinical data of all patients

	Overall	Short-term outcome			Long-term outcome		
	*n* = 160 (%)	*n* = 114 (%)			*n* = 160 (%)		
		Passage	Non-passage	*p* value	Passage	Non-passage	*p* value
		*n* = 49	*n* = 65		*n* = 97	*n* = 63	
Age (yrs): mean ± SD	51 ± 15	50 ± 14	53 ± 15	0.3	51 ± 15	52 ± 15	0.3
Sex				0.4			0.3
Male	112 (70%)	35 (71%)	42 (65%)		71 (73%)	41 (65%)	
Female	48 (30%)	14 (29%)	23 (35%)		26 (27%)	22 (35%)	
Side				0.8			0.3
Right	75 (47%)	23 (47%)	32 (49%)		42 (43%)	33 (52%)	
Left	85 (53%)	26 (53%)	33 (51%)		55 (5%)	30 (48%)	
CRP (mg/L): median (IQR)	2.1 (1.0–7.0)	2.3 (1.0–7.0)	1.8 (1.0–7.7)	0.6	2.3 (1.0–7.0)	1.8 (1.0–7.6)	0.9
	6 missing	2 missing	4 missing		2 missing	4 missing	
MET	59 (37%)	13 (27%)	23 (35%)	0.3	37 (38%)	22 (35%)	0.7
Spontaneous passage	97 (61%)						
Intervention	61 (38)						
No SSP or intervention	2 (1%)						

*SD* standard deviation, *IQR* Interquartile range, *CRP* C-reactive protein, *MET* medical expulsive therapy

**Table 2:** Comparison of radiological parameters between the stone passage and non-passage groups

	Overall	Short-term outcome			Long-term outcome		
	n = 160 (%)	n = 114 (%)			n = 160 (%)		
		Passage	Non-passage	*p* value	Passage	Non-passage	*p* value
		n = 49	n = 65		n = 97	n = 63	
Stone length (mm): median (IQR)	5.7 (4.5–7.3)	4.6 (3.9–5.5)	7.1 (5.8–9.0)	< 0.001	4.8 (4.0–5.7)	7.1 (3.9–5.6)	< 0.001
UWT (mm): median (IQR)	2.4 (1.9–3.3)	2.2 (1.8–2.8)	2.7 (2.2–4.0)	0.003	2.3 (1.9–2.7)	2.9 (2.1–4.1)	< 0.001
Rim sign	25 (16%)	7 (14%)	16 (25%)	0.239	9 (9%)	16 (25%)	0.008
UAAS (HU): mean ± SD	18 ± 13	18 ± 12	15 ± 9	0.196	19 ± 15	17 ± 10	0.1
UABS (HU): mean ± SD	24 ± 15	27 ± 16	23 ± 14	0.088	24 ± 16	24 ± 15	0.2
UDAS (mm): median (IQR)	7.5 (6.3–9.8)	6.6 (8.5–12.2)	9.5 (7.6–12.1)	< 0.001	6.6 (5.9–7.9)	9.9 (8.0–12.1)	< 0.001
UDBS (mm): median (IQR)	6.3 (5.3–7.7)	6.0 (5.1–6.6)	7.1 (6.1 – 8.7)	< 0.001	6.6 (5.9–7.9)	7.1 (6.0–8.7)	< 0.001
RPD (mm): mean ± SD	17.9 ± 6.6	16.9 ± 5.5	19.6 ± 6.5	0.293	16.4 ± 6.0	20.2 ± 6.7	0.1
Hydronephrosis							
Grade 3	28 (17%)	3 (6%)	16 (25%)		12 (12%)	16 (25%)	
Grade 2	82 (51%)	31 (63%)	30 (46%)		50 (52%)	32(51%)	
Grade 1	41 (26%)	9 (19%)	18 (28%)		27 (28%)	14 (22%)	
None	9 (6%)	6 (12%)	1 (1%)		8 (8%)	1 (2%)	

*SD* standard deviation, *IQR* interquartile range, *UWT* ureteral wall thickness, *HU* Hounsfield units, *UAAS* ureteral attenuation above the stone, UABS ureteral attenuation below the stone, *UDAS* ureter diameter above the stone, *UDB*S ureter diameter below the stone, *RPD* renal pelvis diameter

### ROC analysis

Table 3 presents the AUCs for the prediction of SSP with a 95% confidence interval (CI) for stone length and each of the different RSSI parameters. In both the short-term and long-term follow-up, stone length had the highest AUC (AUC: 0.90 and 0.89) followed by UDAS (AUC: 0.85 and 0.82) and UDBS (AUC: 0.73 and 0.69). UWT and RPD showed only low to moderate prediction accuracy (AUC: 0.67/0.66 and 0.62/0.57 (short/long term)), respectively, whereas UABS and UAAS did not predict SSP at all.

**Table 3:** AUC for the prediction of the spontaneous passage of a ureteral stone with different measurements – sub-grouped according to follow-up time

	Short-term outcome			Long-term outcome		
Parameters	AUC	95% CI		AUC	95% CI	
Stone length	0.90	0.84	0.96	0.89	0.85	0.90
UDAS	0.85	0.78	0.92	0.82	0.76	0.85
UDBS	0.73	0.63	0.82	0.69	0.61	0.73
UWT	0.67	0.57	0.76	0.66	0.57	0.67
RPD	0.62	0.52	0.73	0.66	0.57	0.62
UABS	0.41	0.30	0.53	0.48	0.39	0.41
UAAS	0.40	0.29	0.51	0.43	0.34	0.40

*UWT* ureteral wall thickness, *HU* Hounsfield units, *UAAS* ureteral attenuation above the stone, *UABS* ureteral attenuation below the stone, *UDAS* ureter diameter above the stone, *UDBS* ureter diameter below the stone, *RPD* renal pelvis diameter, *CI* confidence interval

**Fig. 4:**
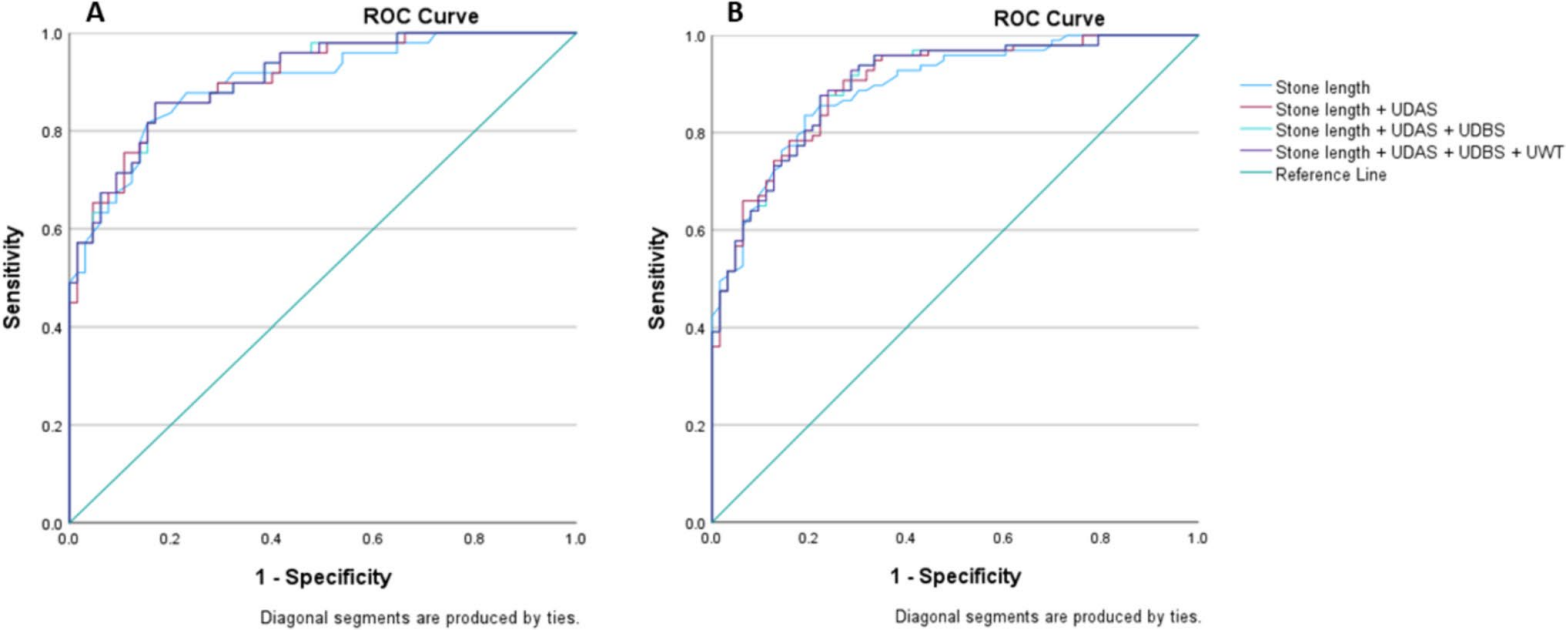
ROC curves for the outcome SSP in the short term (**A**) and long term (**B**): explanatory variables are: stone length either alone or in combination with UDAS, UDBS and UWT; the *y*-axis represents sensitivity, and the *x*-axis shows 1-specificity (*UDAS* ureteral diameter above stone, *UDBS* ureteral diameter below stone, *UWT* ureter wall thickness)

To estimate whether RSSI provided any additional value to stone length in predicting SSP, combined models were computed by the stepwise addition of each of the selected RSSI (UDAS, UDBS and UWT) and calculating the AUCs (Table 4). Figure 4 shows the ROC curves for these combined models. None of the combinations showed a significant increase in prediction accuracy (AUC: 0.90 vs. 0.91 for stone length alone and for stone length + UDAS + UDBS + UWT, respectively).

**Table 4:** AUC(s) for stone length alone and in combination with UDAS, UDBS and UWT, including 95% CI for short-term and long-term outcomes

	Short-term outcome			Long-term outcome		
Parameters	AUC	95% CI		AUC	95% CI	
Stone length	0.90	0.84	0.96	0.89	0.84	0.94
Stone length + UDAS	0.91	0.85	0.96	0.90	0.85	0.95
Stone length + UDAS + UDBS	0.91	0.86	0.96	0.90	0.85	0.95
Stone length + UDAS + UDBS + UWT	0.91	0.86	0.96	0.90	0.85	0.95

*AUC* area under the curve, *CI* confidence interval, *UDAS* ureteral diameter above stone, *UDBS* ureteral diameter below stone, *UWT* ureter wall thickness

### Correlations

We found a high correlation between UDAS and UDBS and both stone length and UWT (|*r*| = 0.7). In addition, collinearity diagnostics revealed the presence of multicollinearity regarding UD measures with VIF values > 5 when checking for all continuous variables.

### Multivariable logistic regression

Stepwise multivariable logistic regression was performed, and the results are summarised in Table 5. Due to multicollinearity, UD variables (UDAS and UDBS) were removed from the multivariable analysis prior to stepwise regression.

**Table 5:** Results of stepwise multivariate logistic regression with spontaneous stone passage as dependent variable and stone length, UWT, rim sign and hydronephrosis as independent variables, odds ratio (OR) for stone passage with 95% CIs

	Short-term outcome					Long-term outcome				
	B	OR	95% CI		*p*	B	OR	95% CI		*p*
Stone length	− 1.29	0.27	0.16	0.47	< 0.001	− 1.14	0.32	0.22	0.47	< 0.001
UWT	− 0.88	0.41	0.18	0.96	0.04	n/a	n/a	n/a	n/a	n/a
Rim sign	− 1.86	0.15	0.02	1.27	0.08	0.66	1.94	0.56	6.74	0.30
Hydronephrosis (ref. none)					0.02					0.39
Hydronephrosis grade (1)	− 5.37	0.00	0.00	0.28	0.01	− 2.13	0.12	0.01	2.34	0.16
Hydronephrosis grade (2)	− 3.97	0.02	0.00	0.96	0.05	− 1.52	0.22	0.01	4.19	0.31
Hydronephrosis grade (3)	− 5.73	0.00	0.00	0.31	0.01	− 1.19	0.30	0.01	7.11	0.46

*B*: regression coefficient, *OR* odds ratio (an OR close to 1 indicates that the variable does not affect the probability of spontaneous stone passage, OR > 1 indicates that this variable is associated with higher probability, and OR < 1 shows that this variable is associated with lower probability of spontaneous stone passage), *CI* confidence interval, *UWT* ureteral wall thickness (mm)

In short-term follow-up, after correcting for other variables, stone length, UWT and hydronephrosis grade were significant predictors for SSP. The rim sign also approached significance in this analysis. Regarding long-term outcomes, only stone length was an independent predictor for SSP.

### Reproducibility of UWT measurements

**Fig. 5:**
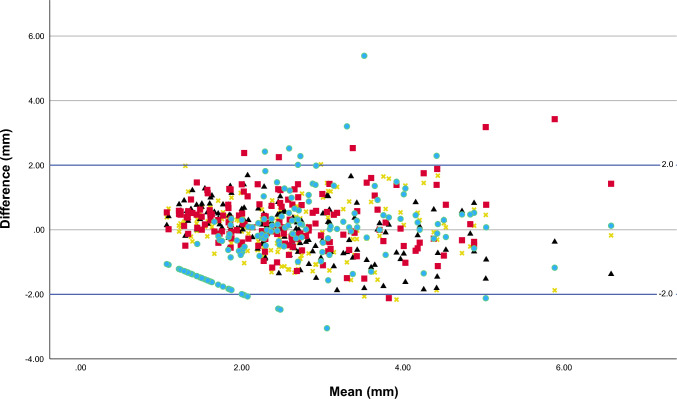
Agreement plot for UWT measurements in millimetres (the five readers are represented by different colours and symbols; horizontal lines indicate the upper and lower limits of agreement with the mean and a line of zero difference); the *x*-axis represents the mean value of all observers’ measurements for each study subject, and the *y*-axis represents the difference between the x̄ and the individual observer’s measurement for each study subject

The ICC was 0.63 (95% CI: 0.52–0.72), showing moderate reliability. Based on the ANOVA test, there was evidence of a systematic difference between the readers (F [1.395] = 20.9, *p* < 0.001). However, the means of each reader were relatively similar, with consistent SDs (Table 6). The estimated limits of agreement (LOA) with the mean was –2.0 to + 2.0 mm, showing that individual observers could be discordant with the mean estimated UWT by 2.0 mm. Agreement plots with the estimated LOA with the mean are presented in Fig. 5. There appears to be a tendency to better agreement with the mean in lower UWT and poorer in higher UWT.

**Table 6:** Distribution of UWT measurements in millimetres for each of the four readers

Observer	Mean (SD) (mm)	Minimum (mm)	Maximum (mm)
1	2.6 (1.2)	0.9	6.4
2	2.7 (0.9)	1.1	5.7
3	2.9 (1.4)	0.8	9.3
4	2.4 (1.9)	0.0	8.9

## Discussion

The main goal of this retrospective study was to investigate the value of several recently proposed ureter-related radiological parameters indicating stone impaction (i.e. UWT, UAAS, UABS, UDAS and UDBS) in predicting spontaneous stone passage. We also aimed to investigate the reproducibility of UWT measurements with the proposed methodology.

Previous research has demonstrated that the size of a stone plays a crucial role in predicting its spontaneous passage [6]. For this study, we utilised the length of the stone, which is defined as the largest diameter measured in one of the three main standard reformats (axial, coronal and sagittal). This diameter was chosen for its ease of measurement and reproducibility. Our results showed that stone size is an excellent predictor, achieving an AUC of 0.90.

Tran et al. and Deguchi et al. recently reported an association between UAAS and UABS and stone impaction, which, in their studies, was verified during ureteroscopy [8, 20]. Moreover, Kachroo et al. stated that UAAS was an independent predicting factor for SPP [12]. Yet, in our material, we could not find any associations between UAAS and UABS and stone expulsion rates.

Despite UDAS and UDBS being significantly larger in the non-passage group compared with the passage group, these measures were highly correlated with the stone length and thus may be simply interpreted as its direct consequence. Therefore, UD measurements did not provide any further predictive value beyond stone length.

Several studies have identified UWT as an independent predictor of SSP, albeit with widely varying cut-off values [11, 14, 15]. The substantial variations in thresholds between the reports likely reflect the heterogeneity of the methods of measurement used across the studies, and the results of these studies have not yet been externally validated. According to a recent systematic review and meta-analysis, increased stone expulsion rates were seen in patients with lower UWT [16].

We could not entirely confirm these results. Although UWT was significantly thicker in the non-passage group both the short term (2.7 mm vs. 2.2 mm, *p* = 0.003) and long term (2.9 mm vs. 2.3 mm, *p* < 0.001), in the multivariable analysis, we found an association between UWT and SSP only in the short-term follow-up. In addition, the prediction accuracy was poor, with AUC = 0.6—considerably lower than the value of 0.88 recently reported by Selvi et al. [17]. One factor that may contribute to the discordant results of this study compared with those of other reports are differences in follow-up times. Most of the earlier published studies measured the outcomes after 4 weeks [11, 12, 14, 15, 18], which might be considered a rather short time, given that—for a ureteric stone > 2 mm—passage may take as long as 40 days [19]. Although the follow-up protocol in our study was not standardised, given its retrospective nature, one strength of this study was its long-term follow-up time of up to 24 weeks, which reflected the natural course of ureteral stones (without intervention).

UWT was a statistically significant predictor for stone passage in the multivariable analysis; however, similar to the UD measurements, it did not add any actual predictive value to that of the stone length. This was confirmed in an ROC analysis in which the RSSI parameters (UDAS, UDBS and UWT) were combined with stone length in a stepwise fashion. We found that the addition of RSSI only increased the prediction accuracy for SSP from 0.90 to 0.91 compared with stone length alone (Table 4, Fig. 4).

To the best of our knowledge, this is the first study addressing the inter-reader variability and reliability of UWT measurements. A reproducibility analysis showed a wide LOA with a mean of − 2.0 to + 2.0 mm for different observers and only low-to-moderate reliability (ICC = 0.63), due to high inter-reader variance. There was also evidence of systematic differences between the readers. On the agreement plot, there was a tendency for better agreement with the mean at lower UWT and worse agreement at higher UWT. These findings indicate that the measurements of UWT on NCCT appear inconsistent and can lead to incorrect interpretation, which questions its usefulness in clinical praxis.

This study has some limitations. Due to its retrospective nature, the follow-up was not standardised regarding either the type of examination or the time after clinical onset. We could, however, identify a subgroup in which the first control of stone status was performed within approximately 4 weeks. The most common follow-up imaging was IVUs, which reflected the clinical routine at our department at that time. Small stones that are radiolucent or have low radiopacity, and cause no obstruction, may be missed on IVU. We estimate, however, that this potential risk for misclassification was rather low and should not significantly affect the results. Having several observers contributed to reducing the observer bias and enhancing data quality, which increased the study’s reliability and validity. However, all the measurements were taken at the same time on different reformations, which could lead to biased results (increased collinearity) due to readers’ objectivity being affected by auto-suggestion and inherent subjectivity.

Of all the investigated ureter-related factors, only UWT independently predicted stone expulsion in short-term follow-up. Still, its accuracy as a single predictor was only low to moderate. Moreover, the UWT measurements exhibited large interobserver variability and low reliability. None of the included stone impaction markers added any significant value to stone length in the prediction accuracy of spontaneous stone passage.

In conclusion, stone size is an excellent predictor for the spontaneous passage of upper ureteral stones. However, radiological signs of stone impaction do not add any clinically significant value as predictors.

## Data Availability

The datasets generated and/or analysed during the current study are not publicly available due to current data protection legislation, but are available from the corresponding author on reasonable request, if appropriate permits are obtained from adequate authorities.

## Abbreviations

SSP: Spontaneous stone passage
RSSI: Radiological signs of stone impaction
UWT: Ureteral wall thickness
UDAS: Ureter diameter above the stone
UDBS: Ureter diameter below the stone
UAAS: Ureter attenuation above the stone
UABS: Ureter attenuation below the stone
HU: Hounsfield units
NCCT: Non-contrast enhanced computed tomography
